# Necessity of Periodic Ophthalmological Examinations in Binocular B Class Driving Licence Holders Over 50 Years of Age

**DOI:** 10.4274/tjo.76258

**Published:** 2016-04-05

**Authors:** Ali Kurt, Çağlar Öktem, Ayşe Karabıçak Acer, Özkan Kocamış, Sedat Taşdemir

**Affiliations:** 1 Ahi Evran University Training and Research Hospital, Department of Ophthalmology, Kırşehir, Turkey

**Keywords:** Binocular B class driving license, individuals over the age of 50, periodic ophthalmological examination

## Abstract

**Objective:**

To determine whether binocular B class driving licence (BBCDL) holders over 50 years old are in compliance with the BBCDL criteria for visual acuity, to determine the age-based prevalence of ophthalmological disorders reducing visual acuity in this group, and to investigate whether periodic ophthalmological examinations are needed in licence holders over 50 years of age.

**Materials and Methods:**

This prospective study enrolled 451 adults over 50 years old having a BBCDL. The study subjects were categorized into 3 age groups as group 1 (51-60 years), group 2 (61-70 years), and group 3 (over 71 years).

**Results:**

The mean age of the subjects was 60.02±7.27 years; 338 (74.9%) were male and 113 (25.1%) were female. The BBCDL criteria were met by 353 (78.3%) subjects whereas 98 (21.7%) subjects did not meet them. Eighty-four (85.7%) of 98 patients not meeting BBCDL criteria still drove. The mean age of the subjects meeting BBCDL criteria (58.82±6.77 years) was significantly lower than the subjects not meeting them (64.34±7.40 years) (p<0.001). The most common pathologies in the individuals still driving despite not meeting BBCDL criteria were senile cataract (38.5%) and diabetic retinopathy (23.1%) in group 1, senile cataract (55.3%) and diabetic retinopathy (14.9%) in group 2, and senile cataract (63.6%) and senile macular degeneration+senile cataract (18.2%) in group 3.

**Conclusion:**

More than a fifth of individuals over 50 years old did not meet the BBCDL criteria, due predominantly to senile cataract, and the majority of these individuals continue to drive. Therefore, we believe that individuals over 50 years old who have a BBCDL should undergo periodic ophthalmological examinations.

## INTRODUCTION

Good vision is crucial for safe driving performance. The proportion of older individuals in the population is increasing, and visual acuity decreases with age. It has been reported that visual screening in older drivers is important for preventing traffic accidents.^1,2^ Aging is accompanied by a higher incidence of sight-reducing conditions such as senile cataract, age-related macular degeneration (AMD) and diabetic retinopathy.^3,4,5^ Diabetic retinopathy in particular progresses with disease duration and requires close follow-up.^6^ The purpose of the current study was to determine whether the visual acuity of individuals over 50 years old with a binocular B class driving licence (BBCDL) is in compliance with the BBCDL criteria, to determine the prevalence of sight-limiting ocular diseases according to age group, and to assess whether periodic ophthalmologic examinations are necessary in this population.

## MATERIALS AND METHODS

For this prospective study, 451 BBCDL holders over 50 years old who presented to the ophthalmology clinic for various ocular complaints between 1 April 2014 and 15 August 2014 were enrolled consecutively. The study adhered to the Declaration of Helsinki and received approval from the local ethics committee. The subjects’ age, gender, and vehicle use were recorded. Visual acuity was measured using the Snellen chart. BBCDL visual acuity requirements are regulated by the bylaw on health status and medical examination of prospective drivers and drivers entered into force through publication in the Official Gazette of the Republic of Turkey (date 26.09.2006, publication number 26301). The bylaw states that drivers receiving a B class licence must have a corrected or uncorrected visual acuity of at least 12/10 total in both eyes, with neither eye less than 2/10. A routine ophthalmologic examination was performed including intraocular pressure measurement and anterior and posterior segment examinations. Subjects were categorized into three groups based on age: group 1, 51-60 years old; group 2, 61-70 years old; group 3, over 71 years old.

### Statistical Analysis

Statistical Package for the Social Sciences version 20 (SPSS, IBM, USA) software was used for statistical analyses. Descriptive statistics were expressed as mean ± standard deviation. Qualitative data were analyzed using the chi-square test. The Mann-Whitney U test was used to compare parameters with non-normal distribution between groups. P<0.05 was considered significant.

## RESULTS

The mean age of the subjects was 60.02±7.27 years (range, 51-82 years); there were 338 (74.9%) males and 113 (25.1%) females. Most of the subjects (78.3%, n=353) were in compliance with the BBCDL criteria for visual acuity, while 21.7% (n=98) did not meet the criteria (Table 1). Although 14 (14.3%) of the 98 noncompliant subjects did not drive, the remaining 84 (85.7%) continued to drive. It was noted that 47 of the 84 subjects who did not meet the BBCDL criteria but continued driving were in group 2 (61-70 years old) (Table 2). The mean age of the compliant subjects (58.82±6.77 years) was significantly lower than that of the subjects who were not in compliance with the BBCDL vision criteria (64.34±7.40 years, p<0.001).

The most common pathologies in subjects not compliant with the BBCDL vision criteria were senile cataract (35.5%), diabetic retinopathy (22.6%), amblyopia and choroidal rupture (9.7%) in group 1; senile cataract (56.0%), diabetic retinopathy (14.0%) and AMD+senile cataract (6.0%) in group 2; and senile cataract (58.8%), AMD (11.8%) and AMD+senile cataract (11.8%) (Table 3).

The most common pathologies in subjects still driving despite noncompliance with the BBCDL vision criteria were senile cataract (38.5%), diabetic retinopathy (23.1%), choroid rupture (11.5%) in group 1; senile cataract (55.3%), diabetic retinopathy (14.9%), and AMD+senile cataract (6.4%) in group 2; and senile cataract (63.6%), AMD+senile cataract (18.2%), posterior capsule opacity (9.1%) and branch retinal vein occlusion (9.1%) in group 3 (Table 4).

## DISCUSSION

The incidence of sight-reducing diseases rises with increasing age,^7,8^ particularly senile cataract, glaucoma, AMD.^3,4,5,9^ Even in the absence of ocular disease, visual function deteriorates with age.^8,10^ A Turkish study comparing ophthalmologic examinations at the age of driving licence acquisition with those later in life showed that the frequency of refraction errors increased approximately 5 fold over a mean period of 20 years.^2^ Therefore, the debate about vision and driving privileges should focus on elderly drivers. This topic will gain importance in the coming years due to the increasing elderly population and the subsequent rise in the number of elderly drivers on the road.

The United States of America and European countries require examinations and certain tests at regular intervals for drivers of advancing age in order to maintain the validity of their driving licence. However, there is no such legal obligation in Turkey.

Senile cataract develops in approximately 60% of adults 60 years of age.^11^ Difficulty reading, driving and perceiving detail are among the resulting functional deficiencies.^12^ Owsley et al.^13^ conducted a visual and ophthalmologic assessment of 2,000 drivers over 70 years old and found that senile cataract had the greatest impact on visual acuity in that age group. Isawumi et al.^14^ evaluated the ocular status of 99 commercial vehicle drivers with a mean age of 45.9 years and found cataract as the second most common (24.3%) cause of vision impairment, while Laudańska-Olszewska et al.^15^ found cataract to be the third most common (6%) cause of vision impairment in a study of drivers aged 60 years and older. Consistent with the literature, in the current study the most common cause of low vision and noncompliance with the BBCDL criteria among all age groups was senile cataract.

The best predictor of diabetic retinopathy is the duration of the disease.6 Patients having type 1 diabetes for 5 years or less rarely show any signs of diabetic retinopathy. In contrast, diabetic retinopathy develops in 27% of patients with diabetes for 5-10 years and 71-90% of patients with diabetes for more than 10 years. After 20-30 years the incidence increases to 95%, and 30-50% of these patients develop proliferative diabetic retinopathy.16 Yanko et al.17 reported the prevalence of diabetic retinopathy in type 2 diabetic patients as 23% at 11-13 years after the onset of type 2 diabetes and 60% at 16 or more years after onset. At 11 or more years after the development of type 2 diabetes, 3% of patients exhibited proliferative diabetic retinopathy. In the current study, the second most common cause of noncompliance with the BBCDL vision criteria in groups 1 and 2 was diabetic retinopathy.

In developed nations, AMD is the most common cause of central vision loss in individuals 65 and older. With a reported frequency of 10% in the 65-74 age range and 25% among those 75 and older, AMD is an important public health issue.18 Vinding19 found that AMD resulted in social blindness (6/60 or lower in both eyes) in 4.3 of every 1,000 people, and monocular blindness in 16.2 of every 1,000 people 60-80 years of age; they also reported that in Denmark, AMD led to visual acuity of 6/9 or lower in one or both eyes of individuals between 60 and 80 years of age. Laudańska-Olszewska et al.^15^ found AMD to be the third most common (7%) cause of vision impairment among drivers 60 years and older. Furthermore, Szlyk et al.^20^ evaluated the driving proficiency of 10 AMD patients with binocular vision of 20/70, and 11 age-matched individuals with normal vision. In the driving simulator test, AMD patients exhibited delayed braking response to stop signals, crossed more into the oncoming lane, and had more simulator accidents. In the current study, the third most common cause of noncompliance with the BBCDL vision criteria in group 2 was AMD+senile cataract, while the second most common cause in group 3 was AMD and AMD+senile cataract.

Visual acuity is easily assessed and is therefore the most commonly measured visual parameter. Although it varies from country to country, binocular vision of 0.5 is accepted as a standard for safe driving in most countries such as European nations and the United States of America (International Council of Ophthalmology at the 30th World Ophthalmology Congress Sao Paulo, Brazil, February 2006). However, in these countries a driving licence is not valid for life. In the United States of America, for example, driving licences have validity periods of several years depending on the state. In most states, driving licences are valid for 4, 5, 6 or 8 years, and in several states, older drivers receive licences with shorter validity periods and/or are subjected to a stricter renewal process. Driving licences are valid for 3 years for individuals over 75 years old in Indiana and for 2 years for drivers over the age of 70 in Iowa. Visual acuity testing is required when renewing a driving licence in Maryland after the age of 40 and in Utah after the age of 65. In Illinois, driving licences are valid for 4 years, and individuals over 75 years old must take a driving test at each renewal. In addition, licences are renewed every 2 years for drivers aged 81-86 and each year after the age of 87 (http://www.ghsa.org/html/stateinfo/laws/olderdriver_laws.html).

According to data compiled by the Turkish Statistical Institute in 2013 including all licence classes, a total of 50,376 drivers (46,309 male, 4,067 female) aged 25-65 were reported injured and 355 (350 male, 5 female) were killed. A total of 2,732 drivers (2,678 male, 54 female) over 65 years old were injured and 60 (59 male, 1 female) lost their lives (Turkish Statistical Institute, Traffic Accident Statistics [Highway] 2013).

Individuals over 50 years old who hold a BBCDL may not have sufficient visual acuity according to the BBCDL specifications, especially due to certain ocular conditions that may occur between 61-70 years of age such as senile cataract, diabetic retinopathy and AMD. In this study, the mean age of patients who were not compliant with the BBCDL vision criteria was statistically higher. Therefore, BBCDL holders over 50 years old should undergo periodic ophthalmologic examinations. Furthermore, we believe that a scientific discussion and revision of the visual function criteria is necessary to improve driver safety in Turkey.

## Ethics

Ethics Committee Approval: Recep Tayyip Erdoğan University Medical School Clinical Research Ethics Board-Date: 05/09/2014 Decision no: 127, Informed Consent: Available.

Peer-review: Externally peer-reviewed.

## Figures and Tables

**Table 1:**
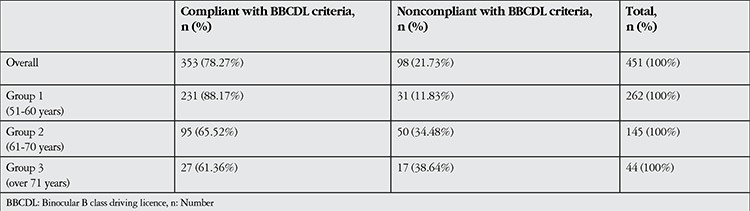
Distribution of binocular B class driving licence vision compliance in subjects overall and within age groups

**Table 2:**
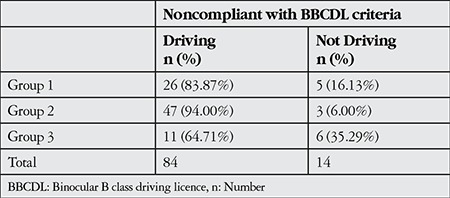
Vehicle usage among subjects not compliant with binocular B class driving licence criteria

**Table 3:**
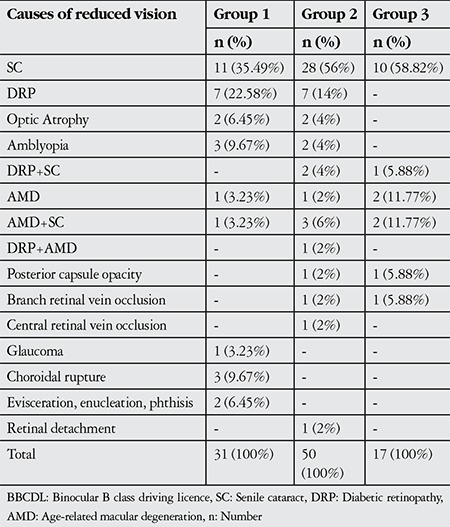
Causes of noncompliance with the binocular B class driving licence vision criteria and their distribution by age group

**Table 4:**
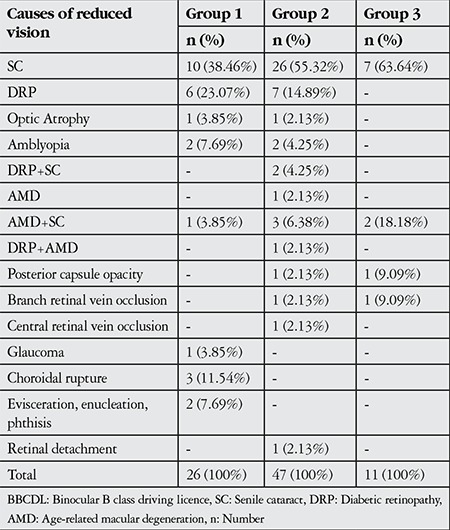
Causes of reduced vision and their distribution by age group in subjects not compliant with the binocular B class driving licence vision criteria who continued to drive
